# Disease duration before surgical resection for chronic pancreatitis impacts long-term outcome

**DOI:** 10.1097/MD.0000000000022896

**Published:** 2020-10-30

**Authors:** Antonie Willner, Andreas Bogner, Benjamin Müssle, Christian Teske, Sebastian Hempel, Christoph Kahlert, Marius Distler, Jürgen Weitz, Thilo Welsch

**Affiliations:** Department of Visceral, Thoracic and Vascular Surgery, University Hospital and Faculty of Medicine Carl Gustav Carus, Technische Universität Dresden, Dresden, Germany.

**Keywords:** chronic pancreatitis, pancreatic surgery, relaparotomy, significant clinical improvement

## Abstract

Many patients with chronic pancreatitis (CP) undergo a step-up approach with interventional procedures as first-line treatment and resection reserved for later stages. The aim of this study was to identify predictive factors for a significant clinical improvement (SCI) after surgical treatment.

All patients operated for CP between September 2012 and June 2017 at our center was retrospectively reviewed. A prospective patient survey was conducted to measure patients postoperative outcome. The primary endpoint SCI was defined as stable health status, positive weight development and complete pain relief without routine pain medication. Additionally, risk factors for relaparotomy were analyzed.

A total of 89 patients with a median follow-up of 38 months were included. In most cases, a duodenum-preserving pancreatic head resection (n = 48) or pancreatoduodenectomy (n = 28) was performed. SCI was achieved in 65.3% (n = 47) of the patients after the final medium follow-up of 15.0 months (IQR: 7.0–35.0 months), respectively. Patients with a longer mean delay (7.7 vs 4 years) between diagnosis and surgical resection were less likely to achieve SCI (*P* = .02; OR .88; 95%CI .80–98). An endocrine insufficiency was a negative prognostic factor for SCI (*P* = .01; OR .15; 95%CI .04–68). In total, 96.2% of the patients had a complete or major postoperative relief with a mean pain intensity reduction from 8.1 to 1.9 on the visual analogue scale.

The results support that surgical resection for CP should be considered at early stages. Resection can effectively reduce postoperative pain intensity and improve long-term success.

## 1 Introduction

Chronic pancreatitis (CP) is a progressive inflammatory disease leading to an irreversible change of the pancreatic parenchyma in fibrotic tissue. Patients suffering from CP have a fivefold higher lifetime mortality and a reduced life expectancy of approximately 8 years less compared to the general population.^[1,2]^ There is abundant evidence that surgical resection (e.g., duodenum-preserving pancreatic head resection [DPPHR] or pancreatoduodenectomy [PD]) is a good treatment option in patients with symptomatic CP.^[3,4]^ In fact, up to 50% of patients without successful endoscopic or interventional therapy require surgical management in the course of their disease.^[5]^ The type of surgical intervention should be tailored individually for each patient based on disease state.^[6]^ Moreover, a PD should be considered if pancreatic cancer cannot be ruled out with certainty.^[4]^ Otherwise, the possibility of parenchyma-sparing resections for preservation of maximum functional pancreatic tissue should be evaluated.^[7]^ In clinical routine, endoscopic interventions are frequently carried out as first-line treatment and surgery is avoided until all other medical and endoscopic treatments have failed repeatedly. Allowing pancreatic pain to persist may lead to years of uncontrollable symptoms and opioid abuse. Many patients suffer from the sequelae of alcohol abuse (e.g., liver cirrhosis) and accumulate significant comorbidities along with disease progression (e.g., portal vein occlusion with porto-venous congestion, ascending cholangitis with hepatic abscesses, cachexia or infected pancreatic pseudocysts) with concomitant increased perioperative risk for surgical intervention. Thus, the timing of operation is an important determinant of long-term clinical outcome in CP.^[8,9]^ It contributes to a long-lasting effect on pain control and improved quality of life (QoL).^[10]^ The most crucial challenge in the management of CP is the early selection of patients who require an operation in order to avoid treatment failure or disease-related complications with concomitant increased perioperative risk.

Thus, the aim of the present retrospective study was to identify factors that can assist treatment allocation. The analysis focused on factors that affected postoperative pain relief, health status, weight development and complications after surgical treatment of CP.

## 2 Patients and methods

### 2.1 Study population and surgical treatment

This retrospective monocentric study was approved by the local institutional review board of the Technische Universität Dresden (decision number: 459112017). All patients with CP scheduled for elective pancreatic resection between September 2012 and June 2017 in the Department for Visceral, Thoracic and Vascular Surgery at the University Hospital Carl Gustav Carus Dresden were identified from a pancreatic database. Patients with confirmed pancreatic cancer in the postoperative histology were excluded.

The type of surgery was tailored to the pathology, symptoms and history. In cases where the CP was mainly limited to the pancreatic head, a DPPHR was attempted. In patients with severe duodenal stenosis or suspected malignancy and the portal vein was accessible, a PD was indicated. The standard approach for the DPPHR was performed according to the Bern modification.^[11]^ The rare presentation of CP limited to the pancreatic tail or remnant after pancreatic head resection was treated by distal pancreatectomy (DP). In cases involving the whole pancreas or recurrent CP, a total pancreatectomy (TP) with or without islet cell auto transplantation (IAT) was performed.

### 2.2 Retrospective data collection

The data were collected and entered in the database created from medical records, surgeons office notes and laboratory parameters. Preoperative clinical characteristics, which could potentially influence the outcome of surgical treatment, were recorded for each patient. These preoperative parameters included patient demographics, body mass index (BMI), etiology of CP, nicotine abuse, laboratory tests (e.g., bilirubin, creatinine, INR for calculation of the MELD score), previous endoscopic interventions, previous surgery and delay from diagnosis of CP until surgical resection.

The preoperative morphology of the pancreatic gland was classified based on the available imaging studies. This presurgical diagnostic workup included computed tomography (CT), magnetic resonance imaging (MRI), magnetic resonance cholangiopancreatography (MRCP), endoscopic retrograde cholangiopancreaticography (ERCP), endoscopic ultrasound and transabdominal ultrasound studies. The imaging modalities were used to assess portal vein thrombosis, portal hypertension, the diameter of the pancreatic main duct and common bile duct, inflammatory pancreatic head enlargement, parenchymal calcification and pseudocystic lesions.

Postoperative events, including morbidity (recorded according to the Clavien Dindo classification of complications [CDC]),^[12]^ formation of postoperative pseudocysts (POPF) and new-onset endocrine insufficiency were also considered. The need for relaparotomy due to major postoperative complications (CDC>3a) was defined as secondary outcome parameter. Separate uni- and multivariate analyses were compiled to address for risk factors regarding the need for relaparotomy.

### 2.3 Clinical improvement and quality of life assessment

Patients included in the retrospective study were interviewed by telephone or seen in the outpatient clinic. The study endpoint “significant clinical improvement” (SCI) was defined as stable health status, positive weight development and complete pain relief without routine pain medication (all 3 items must be fulfilled). The quality of life was further assessed by using the 3-level version of the EQ-5D questionnaire. The EQ-5D-3L descriptive system comprises the following five dimensions: mobility, self-care, usual activities, pain/discomfort and anxiety/depression.^[13]^ In addition, the questionnaire was extended by 4 questions to assess the average pain intensity pre- and postoperatively as well as the ability to reintegrate back into working life and participate in leisure activities after surgery (using a visual analogue scale [VAS]).

### 2.4 Statistical analysis

A statistical analysis was performed using the SPSS software package (SPSS Inc., Chicago, IL) and the graphical representation was realized with GraphPad Prism v7 (GraphPad Software Inc, La Jolla, CA). Due to the retrospective nature of the study, the sample size was not chosen on the basis of power calculations. Data were presented as median values and interquartile range (IQR), unless otherwise indicated. Categorical variables were compared using the Chi-Squared test. After testing for normal distribution, continuous variables were compared using Student *t-*test and ANOVA for normally distributed data and the Wilcoxon or Kruskal–Wallis test for not normally distributed continuous data. Adjustment for multiple testing was not performed. Multivariate analyses were realized using a stepwise backward logistic regression model, adjusting for age, gender, and BMI. Factors from univariate analyses (patient characteristics in Table 1 and operative variables in Table 2) with a *P* value < .05 were included in the multivariate model. *P* < .05 was considered statistically significant. Results were presented as odds ratios (ORs) with 95% confidence intervals (95%CI).

**Table 1:**
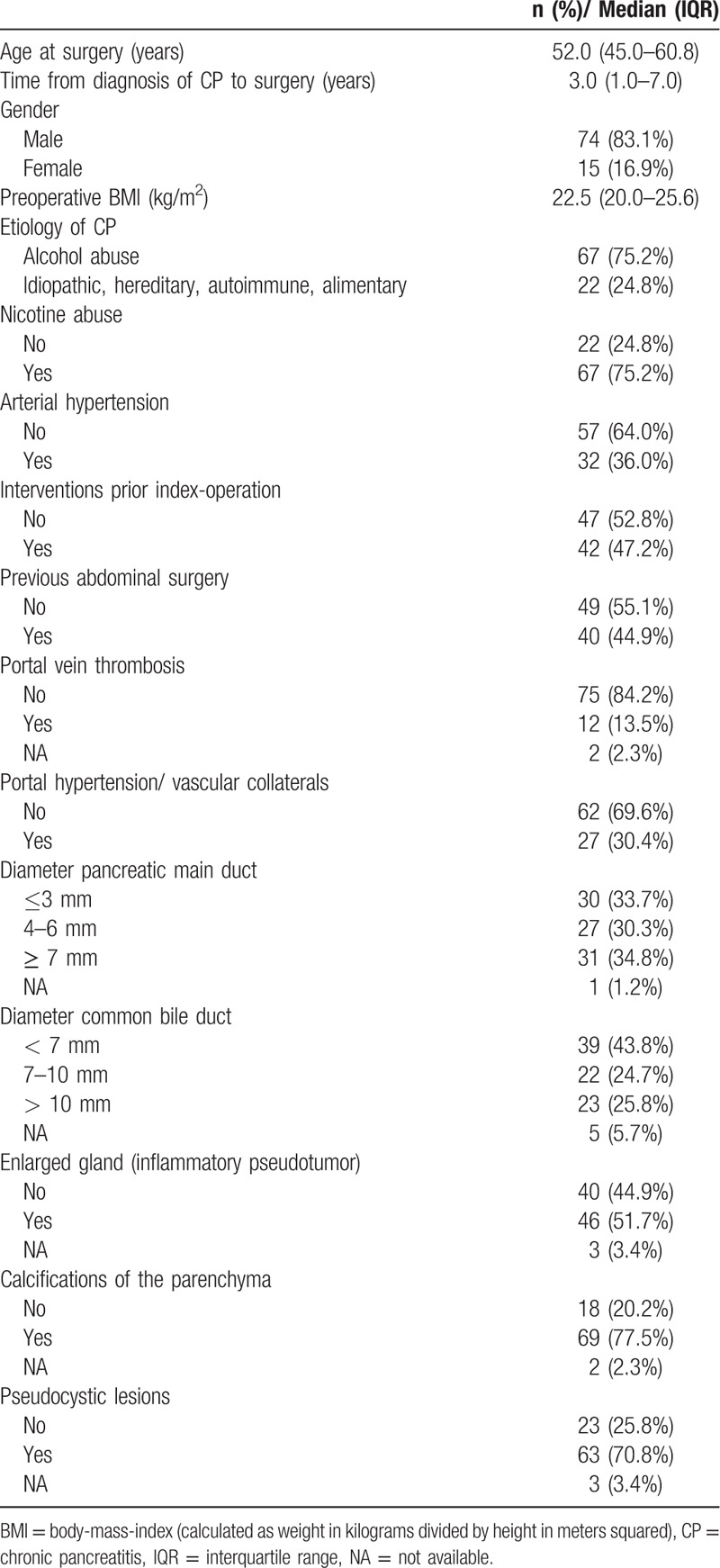
Patient characteristics (n = 89).

**Table 2:**
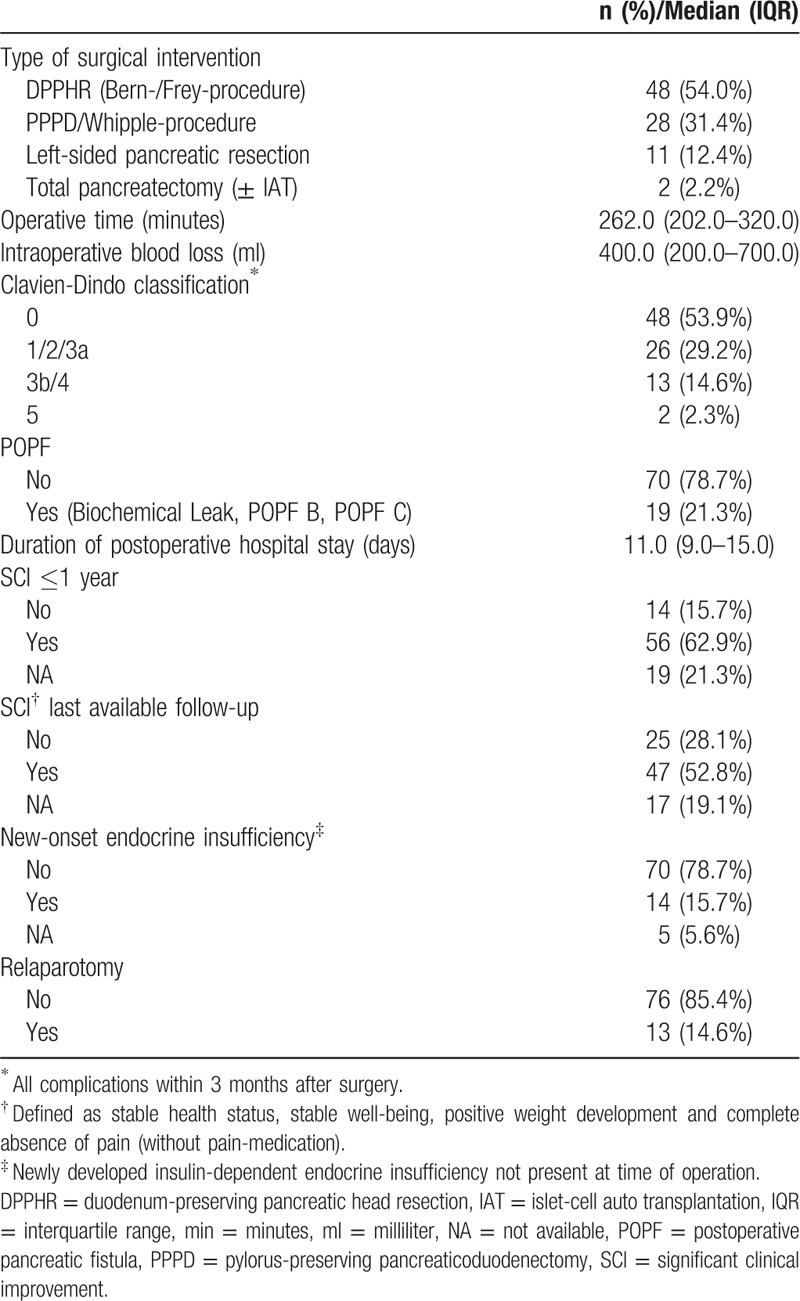
Peri- and postoperative characteristics and patient outcomes (n = 89).

## 3 Results

### 3.1 Patient characteristics and history

In total, 89 patients with a median age of 52 years (83% male) were analyzed (Table 1). The etiology of CP was toxic in 67 (75.2%) patients, whereas idiopathic, hereditary, autoimmune or metabolic causes were identified in 22 (24.8%) patients. Overall, the patient history was positive for chronic nicotine abuse in 75% of cases. A total of 21 patients (23.6%) had a preoperative MELD score of ≥8. The majority of patients reported ≥3 acute episodes of their CP (n = 71, 79.9%) before surgical treatment. The average period between CP diagnosis and pancreatic surgery was 3.0 years (IQR: 1.0–7.0 years). Almost half of the patients have had medical interventions for the treatment of CP before surgery (47.2%) and 45% have had previous abdominal surgeries. The interventions included ERCP (with or without stent intervention) in 29 cases (32.6%), endoscopic transgastric drainage/stenting in 5 cases (5.6%) and endosonography or coiling procedures in 8 cases (9.0%). Of the 40 patients with prior abdominal operations, 16 had prior pancreatic surgery (DPPHR, PD, PPPD or distal pancreatectomy) and 24 had operations independent of the pancreas (e.g., appendectomy, cholecystectomy, adhesiolysis, splenectomy). Portal vein thrombosis or radiological signs of portal hypertension were found in 13.5% and 30.4% of cases, respectively. A marked dilatation of the bile and main pancreatic ducts was seen in 25.8% and 34.8% of cases, respectively.

### 3.2 Perioperative outcome

Most of the patients underwent a DPPHR or pancreatic head resection. A distal or total pancreatectomy (TP) was performed in 13 cases (Table 2). The types of surgical interventions included 48 (53.9%) DPPHRs (Bern procedure: n = 39; Frey procedure: n = 9), 28 (31.5%) PDs (PPPD: n = 24; PD: n = 4), 11 (12.4%) left-sided pancreatic resections, and 2 (2.2%) TPs (with and without IAT). Postoperative morbidity was 46.1% and mortality was 2/89 (2.3%; 90 days after surgery).

Preoperatively, 26 (29.2%) patients had diabetes: 18 patients (20.2%) were insulin dependent, 5 (5.6%) were on oral antidiabetics and 3 (3.4%) were following an antidiabetic diet. Postoperatively, new-onset endocrine insufficiency requiring insulin therapy (Type 3c) was seen in 14 patients (15.7%). The overall POPF rate was 21.3% (Biochemical leak: 7 [7.9%]), Grade B: 8 [9.0%] and Grade C: 4 [4.4%]). A postoperative complication requiring relaparotomy occurred in 13 patients (14.6%) within 90 days after the index operation. The reasons for relaparotomy were: POPF or anastomotic leak (n = 8), hemorrhage (n = 2), pancreatic head necrosis (n = 2), and burst abdomen (n = 1) (Table 2).

### 3.3 Clinical improvement, quality of life and pain relief

According to our definition of a significant clinical improvement (SCI: stable health status, positive weight development and complete pain relief without routine pain medication), a SCI was achieved in 65.3% (n = 47) of patients after the median follow-up period of 15.0 months (IQR: 7.0–35.0 months; n = 72 available patients).

A complete response on the QoL assessment was obtained from 52 (58.4%) patients during follow-up (median: 38.0 months; IQR: 18.5–46.0 months). According to the EQ-5D QoL questionnaire, the majority of patients reported the best outcome in relation to the surgical therapy for all 5 dimensions of the questionnaire: with regard to mobility, self-care and usual activities, 78.9% did not have any problems with ambulating, 88.5% did not have any problems with washing or dressing themselves, and 75.0% did not have any problems doing their usual daily activities. In terms of pain/discomfort, 53.9% had complete pain relief, 42.3% reported moderate pain or discomfort and only 3.8% still reported severe pain. For anxiety/depression, 96.2% were not anxious or depressed after surgery. The median current (postoperative) health status at time of last follow up was reported at a median of 72.5% (scale 0–100%; IQR: 50%–86.3%).

**Figure 1:**
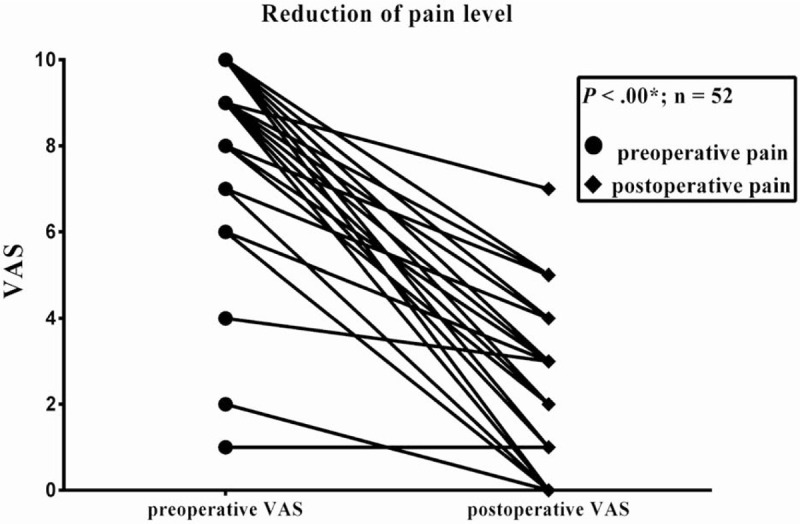
Preoperative pain level in 52 patients is compared to their postoperative level using the VAS (visual analogue pain scale). The median follow-up was 15 months. Data represent pain intensity on a visual analogue scale (0–10) from no pain (0) to the most severe pain (10).

In addition, the patients were retrospectively asked about their pre- and current postoperative pain intensity on a VAS. The patients reported a significant pain reduction from a mean value of 8.1 to 1.9 postoperatively (*P* < .01) (Fig. 1). The average ability of postoperative reintegration into daily social activities (rehabilitation) was scored with 8.0 (IQR: 5.0–10.0; 0 = maximum restriction and 10 = no restriction). At the time of last contact, 29/52 patients in this subgroup (55.8%) were performing their occupational activity. With respect to their postoperative working ability, they reported an average score of 8.0 (IQR: 6.0–10.0). A total of 21 patients were retired and another 3 patients were unemployed because of CP.

### 3.4 Determinants for surgical treatment success

The primary endpoint was SCI after the final follow-up period of the study (15.0 months [IQR: 7.0–35.0 months]). We observed that patients with SCI were of older age (mean age 54.4 years vs 45.1 years; *P* < .01). Importantly, they had a significantly shorter delay between CP diagnosis and surgical treatment (*P* = .04): 4.0 ± 4.8 years (n = 39 patients with SCI; standard deviation ± mean) vs 7.7 ± 6.9 years (n = 21 patients). They also had a lower incidence of postoperative new-onset diabetes (*P* < .01). In multivariate analysis, new-onset diabetes (*P* = .01; OR .15; 95%CI .04–68) and a longer interval between onset of CP and operation (*P* = .02; OR .88; 95%CI .80–98) were inversely correlated with SCI in the long-term (Table 3). A relaparotomy for postoperative complications was another negative predictor for SCI on univariate analysis, only. Thus, the absence of a newly developed diabetes mellitus and a shorter time between diagnosis of CP and operation are linked to a higher likelihood of reaching SCI.

**Table 3:**
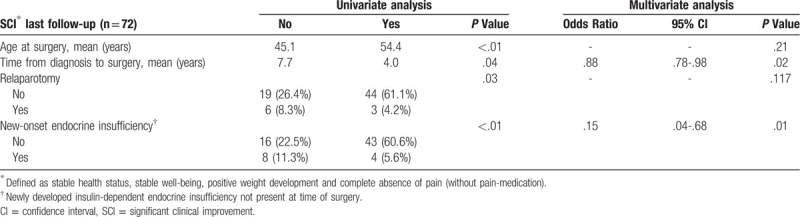
Univariate and multivariate analysis of risk factors associated with a significant clinical improvement (SCI) after surgery.

When analyzing risk factors for postoperative morbidity (CDC>3a), univariate analysis identified operating time (324.5 vs 270.5 minutes; *P* < .00) and length of hospital stay (30.0 vs 13.5 days; *P* < .01) as significant factors for postoperative relaparotomy. The latter can be explained by the complicated postoperative course.

## 4 Discussion

In the last few years, further considerable progress has been made in understanding the development of CP. Many experts in the field consider CP a continuous disease process, evolving from acute pancreatitis (AP) and recurrent AP to early and end-stage CP, as outlined in a recent international consensus draft on a mechanistic definition of CP.^[14]^ This disease progression recognizes the complex and still not fully understood nature of CP. The typical morphological changes, such as calcifications, parenchymal lobulation, atrophy, pseudocysts and pancreatic duct abnormalities, can be made visible. However, there is a lack of predictive markers or markers to identify those patients at risk of disease progression.^[15]^

Since the disease course is often unpredictable, the optimal treatment remains a clinical challenge. In addition, an understanding of the key players in pancreatic inflammation is crucial for improved management. In general, the inflammatory process in the pancreatic head is considered to be the pacemaker of CP. Today, a DPPHR modified according to Beger or Bern, a PD, or pancreatic duct drainage procedures with coring out of the pancreatic head (Frey procedure) are accepted surgical options for treating CP and do not show any differences in quality of life within 24 months after surgery.^[4]^ DPPHR eradicates the pain syndrome in most patients (>85%) and eliminates local complications of CP.^[16]^ The significant pain relief after surgery was also shown in the present analysis. However, surgical resection competes with less invasive endoscopic options. Many centers follow a step-up approach to managing CP, starting with pain medication and escalating from multiple endoscopic interventions (e.g., endoscopic retrograde choledochal or pancreatic duct stenting, or transgastric drains) to surgery at the end of the treatment pathway. Consequently, many patients are referred to a surgeon only after years of symptomatic minimally invasive procedures and a chronic pain memory has been established. Years of opioid abuse and repeated interventions often just treat the symptoms without eliminating the root of the problem.^[17]^ Furthermore, surgical resection is thought to remove the chronic inflammation trigger in the pancreatic head and effectively reduce pain in patients. Delayed surgical treatment can even prevent patients from achieving SCI.^[9]^ In line with this, the present study demonstrated that patients with shorter disease duration until surgery are more likely to achieve SCI. This important finding is supported by clinical trials and was recently included in consensus guidelines.^[9,18]^ Recently, the ESCAPE study results support that early resection within the first months of opioid use, provides better pain relief with fewer interventions than the current step-up approach.^[19]^

According to Kempeneers et al early surgery compared to a long history of multiple endoscopic interventions is associated with a reduced risk of failure concerning pain relief, pancreatic insufficiency and re-intervention rates.^[9]^ We failed to show direct connection between endoscopic interventions and higher risk of reoperation. But we could demonstrate a correlation between patients who underwent relaparotomy and those not to achieve SCI. From a surgical perspective, this can be explained by the more complex anatomical situation requiring high-risk surgery after less invasive procedures.

While redo surgery for CP is demanding, it can be performed with acceptable morbidity in high-volume centers.^[20]^ However, there has been discussion about how inadequate primary surgery for CP significantly influences the rate of redo surgeries. In addition, partial resection of the pancreatic parenchyma with drainage of the choledochal and pancreatic duct seems to be superior to drainage procedures alone with regard to the need for redo surgery.^[21,22]^ According to the latest results of the ChroPac trial, a PD is at least an equal alternative if a substantial subtotal pancreatic head resection cannot be accomplished during a DPPHR.^[4]^ In this field, a standardization of procedures and perioperative treatment is critical. This might enable high-volume centers to significantly reduce mortality and morbidity rates in order to maximize patient safety. Our results show that the risks and mortality rate for these operations are tolerable. In general, however, these procedures still pose a substantial risk, especially with regard to surgical complications. This is something that needs to be discussed individually with the patient. The fact that relaparotomy is associated with lower chance in achieving SCI status in our cohort supports the concept of centralizing treatment for patients with CP in high-volume centers. Higher procedure volume even plays a larger role than increased experience in reducing inpatient death rates.^[23]^ The high costs for health care systems and the burden placed on society due to insufficient treatment, hospital readmissions and long-lasting unemployment have to be considered as well.^[24]^

The present study has some limitations, which need to be considered. First, the study design was retrospective and not randomized, which results in a bias regarding the treatment approach, type of resection and monitoring/follow-up of the patients. Patient and treatment selection therefore had a potential influence on the studys outcome. Second, QoL was not routinely assessed preoperatively and, consequently, a direct comparison of pre- and postoperative QoL scores was not performed. Third, we analyzed a limited patient cohort size and observed a 22.5% dropout rate during the follow-up, which probably influenced the outcome data as well. However, the few available randomized controlled trials (RCT) show analogous results. Nevertheless, more RCTs are needed in order to demonstrate the superiority of early surgery compared to the standard step-up approach. Based on the findings of this study (i.e., earlier surgical resection results in high SCI rates, good pain control and QoL), we believe there is a need for strong interdisciplinary management of CP patients and discussion within gastrointestinal boards at specialized centers. The recently published Chronic Pancreatitis Pain Relief Score by Bachmann et al. might be an additional promising tool to identify those patients who benefit most from surgical procedures.^[25]^

In summary, the present retrospective analysis underlines that the choice to use a routine step-up approach until surgical resection should be critically assessed and surgical therapy of CP should be considered earlier (the timing must be tailored individually according to disease course/activity, comorbidities and symptoms) after diagnosis of symptomatic CP. Multiple interventions over years prior to surgical resection (i.e., an exhausted step-up approach) can probably delay the course of disease, increase the risk of postoperative complications and negatively influence the long-term outcome. On the other hand, adequately performed surgical procedures can effectively reduce pain intensity during long-term follow-up.

## Author contributions

All authors revised the manuscript critically, approved the final version and agreed to be accountable for all aspects of the work.

**Antonie Willner:** patient acquisition, data collection, drafting of the manuscript.

**Andreas Bogner:** data analysis, data interpretation, drafting of the manuscript.

**Benjamin Müssle:** data analysis, design and concept of the study.

**Christian Teske:** data interpretation, design and concept of the study.

**Sebastian Hempel:** data interpretation, design and concept of the study.

**Christoph Kahlert:** data interpretation, design and concept of the study.

**Marius Distler:** data interpretation, design and concept of the study.

**Jürgen Weitz:** data interpretation, design and concept of the study.

**Thilo Welsch:** data interpretation, design and concept of the study, drafting of the manuscript.
